# The determination of adherence to fluid control and symptoms of patients undergoing hemodialysis

**DOI:** 10.4314/ahs.v22i3.38

**Published:** 2022-09

**Authors:** Ali Kaplan, Songül Karadağ

**Affiliations:** 1 Kayseri University, İncesu Ayşe and Saffet Arslan Vocational School of Health Services, Department of Medical Services and Techniques; 2 Çukurova Universitesi, Department of Internal Diseases Nursing, Faculty of Health Sciences

**Keywords:** Hemodialysis, fluid control, adherence, symptom, nursing

## Abstract

**Background and aims:**

The aim of this study was to determine the adherence of hemodialysis (HD) patients with fluid control and the symptoms they experienced.

**Method:**

The data of the study were collected between October 2015 and January 2016 and totally 596 patients undergoing hemodialysis were included in the study. The data of the study were collected by using Patient Information Form, Fluid Control Scale on Hemodialysis Patients (FCSHP), and Dialysis Symptom Index (DSI).

**Results:**

Total mean score patients received from FCSHP was 48.68±4.43, score of the subscale information was 18.85±2.24, score of the subscale behavior was 21.28±3.23, and score of the subscale attitude was 8.54±1.56. Mean score obtained by them from DSI was calculated as 65.07±2.17.

Symptoms that patients experience most frequently were found as feeling tired or decreased energy, pins and needles in feet, and having difficulty in falling into sleep. The patients with high adherence to fluid control were found to have less symptoms.

**Conclusion:**

It was recommended to assess periodically adherence to fluid control in individuals receiving hemodialysis treatment and symptoms they experience and to provide training and consultancy by addressing those having difficulty in adherence to fluid control.

## Introduction

Chronic renal failure (CRF) is a chronic disease that results in reduced glomerular filtration rate (GFR) resulting in progressive deterioration in the metabolic-endocrine funtions and impaired fluid-solute balance^1^. The prevalance of CRF in the World is increasing significantly and the cost of its treatment is a great burden^2^. Considering the prevalance of End Stage Renal Disease (ESRD) that occurs due to the progress of the CRF in the United States Kidney Registry System (USDRS) atlas, 2309 patients in Japan, 1924 patients in USA, 1662 patients in Portugal and 1661 patients in Singapore per million^3^. According to the data of 2016 from the Turkish Society of Nephrology, the prevalance of ESRD requiring renal replacement therapy (RRT) was found to be 993 per million^4^.

Renal replacement therapies such as Continuous Ambulatory Peritonel Dialysis (CAPD), Hemodialysis (HD) and kidney transplantation are applied for the treatment of patients with CRF^5^. Hemodialysis is the most preferred method for the treatment of CRF all over the World. According to the data of 2014, 91.3% in the USA,75.8% in Sweden, 91.6% in Russia, 88.9% in France and 85.0% of CRF patients in Turkey were receiving hemodialysis treatment^6^.

The individual's compliance with the treatment and health recommendations is very important for the success of hemodialysis treatment. As with all chronic diseases, it is vital for individuals receiving HD treatment to comply with drug therapy, diet, and fluid restriction in order to maintain their health^7^. It has been determined that adherence to treatment of individuals receiving HD treatment reduces long-term dialysis complications and helps to prevent and reduce complications that may occur after transplantation^8^.

Fluid restriction is the most stressful situation and makes it difficult for HD patients to comply with treatment^9^,^10^. If the amount of fluid taken between two dialysis is more than 5.7% of the dry weight after dialysis, this indicates inappropriate weight gain. Excessive fluid intake can result in severe complications such as hypervolemia, edema, acid accumulation, left ventricular hypertrophy, congestive heart failure, pulmonary vascular occlusion and acute pulmonary edema^11^. In many studies on the subject, more than half of hemodialysis patients do not comply with fluid restriction^12^,^13^.

The symptoms experienced by HD patients and the problems they experience regarding fluid restriction prevent them from performing their daily activities and cause a decrease in their quality of life^14^. Increasing the quality of life of HD patients is possible with effective evaluation of patients and providing appropriate care for symptoms^15^. Many symptoms may adversely affect the quality of life due to fluid restriction can be seen in HD patients. Hypotension, hypertension, insomnia, cramps, fatigue, weakness, pain, edema, nausea-vomiting, sexual dysfunction, anorexia and anemia are among the most common symptoms of HD patients^16^,^17^. A multidisciplinary team should work for the adherence to the treatment of HD patients^18^. As they are in contact with patients for longer time, the nurses involved in this team play a key role in follow-up and adherence to the treatment of HD patients.

Although the importance of fluid control and symptom management in HD patients is emphasized in the literature, there are not enough studies on this subject^12^,^19^. In order to provide fluid control and symptom management, it is necessary to reveal the current situation as a priority. However, comprehensive and practical studies can be planned for the situation determined in this way. This study was carried out with the aim of determining the compliance of hemodialysis patients with fluid control and the symptoms they experience.

## Materials and methods

### Study design

The population of the study was all the hemodialysis patients followed in hemodialysis unit of a university hospital and 5 private Dialysis Centers in Kayseri, Turkey. Patients who received hemodialysis treatment for at least 6 months and agreed to participate in the study were included in the study. Patients with physical and mental health problems that would prevent communication were not included in the study. All patients who met the inclusion criteria and agreed to participate were included in the study. 85.8% of the population was reached and the study completed with 596 HD patients.

The data of the study were collected between October 2015 and January 2016 and totally 596 patients undergoing hemodialysis (294 women, 302 men) were included in the study. The data of the study were collected through face-to-face interview method. All forms were administered to the patients simultaneously by the investigator during the hemodialysis treatment session.

The data in the study was collected using Patient Information Form, Fluid Control Scale on Hemodialysis Patients (FCSHP) and Dialysis Symptom Index.

Patient Information Form: This form prepared by the researcher in accordance with the literature^16^,^20^ includes the socio-demographic characteristics such as age, occupation, sex, marital status, educational status and disease characteristics such as existence of additional chronic diseases, duration of diagnosis, training on fluid restriction and vital signs.

Fluid Control Scale on Hemodialysis Patients (FCSHP): The scale was developed by Coşar and Pakyüz^21^ in order to evaluate the knowledge, behaviour and attitude about the fluid restriction of HD patients. The scale is a measurement tool that is frequently used nationally and internationally to determine the compliance of hemodialysis patients with fluid control^22^,^23^. There are total of 24 items and 3 subscales on the scale.

1–7^th^ questions are Knowledge Subscale, 8–18th questions are Behaviour Subscale and 19–24^th^ questions are Attitude Subscale.

In the evaluation of likert type scale, there are three items; I agree (3), Undecided (2), I do not agree (1). 1, 2, 3, 4, 5, 8, 9, 10, 11, 12, 13, 14, 15, 16, 17^th^ items are scored positively and 6, 7, 18, 19, 20, 21, 22, 23, 24^th^ items are scored negatively. The lowest score that can be taken from the scale is 24, the highest score is 72. As the score of the scale increased, patients' adherence of fluid control increased^21^. The scale's general Cronbach's alpha internal consistency coefficient was 0.88. In the present study, the Cronbach's alpha coefficient of the overall FCHPS was 0.72.

Dialysis Symptom Index (DSI): The scale was developed by Weisbord et al. in 2004^24^ and validity and reliability was shown by Önsöz and Yeşilbakan^25^. The index consists of 30 items for evaluating physical and emotional symptoms and their severity. The patients replied with “Yes/No” the questions about symptoms one week ago and how these symptoms affected them was questioned. If the answer is “No”, the point is “0”. If the answer is “Yes”, there is a likert type scale (1= did not bother, 2= slightly disturbed, 3= sometimes disturbed, 4= very uncomfortable, 5= very disturbed) to answer. The lowest score that can be taken from the index is 0, the highest score is 150 and as the score increases, the sypmtoms of dialysis also increase. Cronbach's alpha coefficient of the DSI was determined to be 0.83^25^.

Ethical principles were followed at every stage of the study. An approval of the Academic Committee was taken from Erciyes University Health Sciences Institute. Institutional permits received from Dialysis Centers and Ethichs Committee Approval (Decision Number: 2015/443, Date: 02.10.2015) were obtained from Erciyes University Faculty of Medicine Clinical Investigation Ethics Committee. Verbal and written informed voluntary consent form was obtained from all the patients participating in the study. The ethical principles of the Declaration of Helsinki were complied with at all stages of the study.

### Statistical analysis

The data obtained from the study were evaluated using SPSS 16.0. Pearson moment correlation was used for the test-retest analysis and the consistency of the scale and subscales of the scale and sociodemoghraphic data of patients was shown as number, percentage, mean-standart deviation and median. Mann Whitney u test in two dependent group comparisons and Kruskal Wallis test in three or more group comparisons were used for the evaluation of nonparametric data. Samples-t test in two dependent group comparisons, One-Way ANOVA in three or more group comparisons and Post-Hoc (Tukey) test in order to determine the significance between groups were used for the evaluation of parametric data. The value of p<0.05 was considered statistically significant.

## Results

Baseline charecteristics of the patiens were shown in Table 1. 50.7% of the patients were male, 32.9% were in 60–69 age group and the average age was 62.71 among all participants. 72.8% were married, 48.0% were primary school graduate and 56.7% were retired. It was determined that 44.6% of the patients had a diagnosis of CRF for 3–8 years, 37.4% had HD for 2–5 years, 68.3% had information about diet and fluid restriction, and 87.4% had coexisting chronic disease. It was detected that 38.4% of the patients in the study group had daily fluid intake of ≤1000 ml, 40.6% had dry weight of 60–75 kg, and 38.1% had interdialytic weight of 1.5–2.4 kg.

**Table 1:** Descriptive characteristics of patients

Characteristics	*n (%)*	Characteristics	*n (%)*
**Sex**		**HD duration**	
Female	294 (49.3)	< 2 years	125 (21.0)
Male	302 (50.7)	2–5 years 6–9 years ≥ 10 years	223 (37.4) 137 (23.0) 111 (18.6)

**Age group (mean: 62.71)**		**Getting information about diet and fluid** **restriction**	
≤ 49 years	91 (15.3)		
50–59 years	115 (19.3)	Yes	407 (68.3)
60–69 years	196 (32.9)	No	189 (31.7)
≥ 70 years	194 (32.5)		

**Marital status**		**Additional chronic disease**	
Married	434 (72.8)	Available	521 (87.4)
Single	162 (27.2)	Not available	75 (12.6)

**Education status**		**Chronic Disease** (*n*=521)*	
Literate / Illiterate	195 (32.7)	Hypertansion	453 (86.9)
Primary school	286 (48.0)	DM	272 (52.2)
Secondary education	91 (15.3)	Heart Disease	68 (13.0)
Bachelor	24 (4.0)	Pulmonary disease	35 (6.6)

**Job**		**Daily fluid intake**	
Retired	338 (56.7)	< 1000 ml	229 (38.4)
Housewife	221 (37.1)	1000–1500 ml	181 (30.4)
Other (Free, Worker, Officer)	37 (6.2)	> 1500 ml	186 (31.2)

**BMI**		**Dry weight**	
<18.5 kg/m2	26 (4.4)	< 60 kg	142 (23.8)
18.5–24.9 kg/m^2^	205 (34.4)	60 – 75 kg	242 (40.6)
25.0–29.9 kg/m^2^	215 (36.1)	76 – 91 kg	152 (25.5)
>30.0 kg/m^2^	150 (25.1)	≥ 92 kg	60 (10.1)

**CRF diagnosis duration**		**Interdialytic weight**	
< 3 years	110 (18.5)	< 1.4 kg	84 (14.1)
3–8 years	266 (44.6)	1.5–2.4 kg	227 (38.1)
9–14 years	134 (22.5)	2.5–3.4 kg	185 (31.0)
≥ 15 years	86 (14.4)	≥ 3.5 kg	100 (16.8)

* multiple answers

It was found that the mean score of patiens on the DSI scale was 65.07 ± 2.17. The mean total score of the FCSHP scale was 48.68 ± 4.43 and the knowledge subscale was 18.85 ± 2.24; behaviour subscale was 21.28 ± 3.23 and attitude subscale was 8.54 ± 1.56.

Table 2 presents the symptoms that patients experience according to DSI. The most common symptoms of the patients are, tiredness or decreased energy (96.3%), numbness and tingling of feet (71.3%), difficulty in sleeping (65.9%) and maintaining sleepiness (65.3%), respectively. The most severe symptoms were, tiredness or decreased energy (4.9%), numbness and tingling of feet (4.8%), bone or joint pain (4.8%), chest pain (4.8%), muscle pain (4.8%), lack of sexual desire (4.8%) and difficulty in sexual arousal (4.8%), respectively.

**Table 2:** Symptoms of patients' according to DSI

Symptoms	*n*	*(%)*	*Mean Severity*
1. Constipation	319	53.5	4.6
2. Nausea	197	33.1	3.8
3. Vomiting	154	25.8	3.7
4. Diarrhea	72	12.1	3.9
5. Loss of appetite	197	33.1	4.2
6. Muscle cramps	381	63.9	4.7
7. Swelling on the legs	136	22.8	4.4
8. Dyspnea	286	48.0	4.4
9. Drowsiness/Dizziness	256	43.0	4.0
10. Difficulty in keeping legs motionless	102	17.1	4.5
11. Laziness of feet and tingling	425	71.3	4.8
12. Feeling tired and reduced energy	574	96.3	4.9
13. Cough	226	37.9	4.1
14. Dryness of the mouth	243	40.8	4.4
15. Bone or joint pain	324	54.4	4.8
16. Chest pain	227	38.1	4.8
17. Headache	281	47.1	4.7
18. Muscle pain	212	35.6	4.8
19. Difficulty in concentrating	111	18.6	4.1
20. Dryness of the skin	382	64.1	4.6
21. Itching	368	61.7	4.6
22. Beeing worried	311	52.2	4.7
23. Feeling nervous	358	60.1	4.7
24. Difficulty in sleeping	393	65.9	4.6
25. Difficulty in maintaining sleep	389	65.3	4.6
26. Feeling uncomfortable	311	52.2	4.3
27. Feeling sad	323	54.2	4.3
28. Feeling anxious	381	63.9	4.4
29. Decrease in sexual appetite	259	43.5	4.8
30. Difficulty in being stimulated sexually	258	43.3	4.8

Table 3 shows the relationships between total and subscale scores of FCSHP, DSI scale and interdialytic weight of patients. There was a significant negative correlation between DSI and FCSHP knowledge, behaviour subscales and total score (p<0.001). Interdialytic weight had a significant positive correlation with FCSHP knowledge subscale and significant negative weak correlation with behaviour subscale (p<0.001).

**Table 3:** The relationship between FCSHP and DSI scale scores

	FCSHP Knowledge	FCSHP Behaviour	FCSHP Attitude	FCSHP Total	DSI	Total	Interdialytic weight
DSI Total *rho* *p*	-0.241 <0.001	-0.186 <0.001	-0.060 0.142	-0.265 <0.001	-		

Interdialytic weight *rho* *p*	0.158 <0.001	-0.117 <0.001	-0.017 0.673	-0.011 0.782	0.054 0.190		-

According to some characteristics of the patients included in the study, total and subscale scores of FCSHP and DSI scores are shown in Table 4. FCSHP total and knowledge subscale scores were significantly higher in male and ≤49 age HD patients (p<0.001). It was found that median FCSHP knowledge subscale score was higher in patients with normal BMI (p<0.05). Median FCSHP total and knowledge subscale scores were sifnificantly higher in married and bachelors's degree patients (p<0.001). The FCSHP knowledge, behaviour subscales and total scores were higher than those of retired and housewives, and this difference was found to be statistically significant (p<0.05). The FCSHP knowledge subscale and total scores were found to be higher inworking patients compared to those who did not work (p<0.05).

**Table 4:** FCSHP and DSI scores according to some characteristics of patients

Characteristics	FCSHP Knowledge *Median (Q_1_-Q_3_)*	FCSHP Behaviour Median (Q_1_-Q_3_)	FCSHP Attitude Median (Q_1_-Q_3_)	FCSHP Total Median (Q_1_-Q_3_)	DSI Total X ± *SS*
**Sex** Female Male	18.0 (16.0–21.0) 21.0 (19.0–21.0)	22 (19.0–24.0) 21 (19.0–23.0)	9 (8.0–9.0) 9 (8.0–10.0)	49 (45.0–51.0) 49 (46.0–52.0)	68.29 ± 22.09 61.94 ± 20.93

*p*	**<0.001**	0.087	0.772	**0.049**	**<0.001**

**Age** ≥49 years 50–59 years 60–69 years ≥70 years	21.0 (20.0–21.0)^a^ 21.0 (19.0–21.0)^a^ 19.0 (17.0–21.0)^b^ 18.0 (16.0–20.0)^c^	21.0 (19.0–24.0) 21.0 (19.0–24.0) 22.0 (20.0–23.0) 21.0 (19.0–23.0)	9.0 (8.0–10.0) 9.0 (8.0–9.0) 9.0 (8.0–9.0) 9.0 (8.0–9.0)	50.0 (48.0–53.0)^a^ 50.0 (47.0–52.0)^a^ 49.0 (46.0–52.0)^a^ 48.0 (44.0–51.0)^b^	62.54 ± 24.01 66.80 ± 23.28 64.67 ± 22.29 65.63 ± 18.95

*p*	**<0.001**	0.573	0.377	**<0.001**	0.541

**BMI** <18.5 kg/m^2^ 18.5–24.9 kg/m^2^ 25.0–29.9 kg/m^2^ >30.0 kg/m^2^	19.0 (17.0–21.0)^a,b^ 21.0 (17.5–21.0)^a^ 19.0 (17.0–21.0)^a,b^ 19.0 (17.0–21.0)^b^	21.0 (18.7–23.0) 21.0 (19.0–23.0) 21.0 (20.0–23.0) 21.0 (19.0–24.2)	9.0 (7.7–10.0) 9.0 (8.0–10.0) 9.0 (7.0–9.0) 9.0 (8.0–9.0)	49.0 (46.0–51.2) 50.0 (46.0–52.0) 49.0 (46.0–52.0) 48.0 (45.0–51.0)	66.30 ± 20.13 61.56 ± 22.53 66.77 ± 21.45 67.23 ± 20.86

*p*	**<0.001**	0.547	0.318	0.479	**0.049**

**Marital status** Married Single	20.0 (17.0–21.0) 18.0 (16.0–21.0)	21.0 (19.0–23.0) 21.0 (19.0–23.0)	9.0 (8.0–9.0) 9.0 (8.0–9.0)	49.0 (46.0–52.0) 48.0 (44.0–51.0)	65.85 ± 21.79 62.97 ± 21.47

*p*	**<0.001**	0.651	0.950	**<0.001**	0.150

**Education status** Literate / Illiterate Primary school Secondary education Bachelor	17.0 (16.0–19.0)^a^ 19.0 (17.0–21.0)^b^ 21.0 (19.0–21.0)^c^ 21.0 (21.0–21.0)^c^	22.0 (19.0–24.0) 21.0 (19.0–23.0) 21.0 (19.0–23.0) 23.0 (21.0–26.5)	9.0 (8.0–9.0) 9.0 (8.0–9.0) 9.0 (8.0–10.0) 8.5 (8.0–10.0)	48.0 (45.0–51.0)^a^ 49.0 (46.0–52.0)^a^ 50.0 (47.0–53.0)^b^ 53.0 (50.0–54.7)^b^	68.93 ± 20.83 63.93 ± 21.88 62.87 ± 21.40 55.62 ± 23.96

*p*	**<0.001**	0.651	0.950	**<0.001**	0.150

**Job** Retired Housewife Working	20.0 (17.0–21.0)^a^ 19.0 (17.0–21.0)^b^ 21.0 (18.0–21.0)^a^	21.0 (19.0–23.0)^a^ 22.0 (19.0–24.0)^b^ 23.0 (21.0–25.0)^a^	9.0 (8.0–9.0) 9.0 (8.0–9.5) 8.0 (6.5–10.0)	49.0 (46.0–52.0)^a^ 49.0 (46.0–52.0)^a^ 51.0 (47.0–54.0)^b^	63.79 ± 20.14^a^ 68.70 ± 22.91^b^ 55.10 ± 24.49^a^

*p*	**<0.001**	**<0.001**	0.159	**0.029**	**<0.001**
**Working status** Working Not working	21.0 (21.0–21.0) 19.0 (17.0–21.0)	23.0 (21.0–25.0) 21.0 (19.0–23.0)	8.0 (7.2–9.7) 9.0 (8.0–9.0)	53.0 (48.0–54.7) 49.0 (46.0–52.0)	52.25 ± 21.78 65.42 ± 21.64

*p*	**<0.001**	0.086	0.529	**0.010**	**0.017**

a,b,c: Comparisons between measurements in each group

When the DSI scores of the patients included in the study were examined, it was found that the DSI scores of the women, obese and housewives had significantly higher scores than the other patients (p<0.001).

Table 5 shows the FCSHP and DSI scale scores of patients according to the characteristics of their coexisting diseases. Patients without additional chronic disease had higher FCSHP knowledge subscale and total scores, and this difference was statistically significant (p<0.001). Patients having 3–8 years of CRF diagnosis were found to have higher FCSHP knowledge subscale score than others (p<0.05). Patients with a duration of HD less than 2 years had higher FCSHP behaviour subscale score and this difference was also statistically significant (p<0.01). Patients who had information about diet and fluid restriction and daily fluid intake of ≥1500 ml had higher FCSHP knowledge subscale and total scores (p<0.05). Mean DSI scores were higher in the patients with additional chronic disease than others, and this difference was statistically significant (p<0.001). The mean DSI scores of patients receiving HD treatment for ≥10 years were higher than other groups (p<0.05).

**Table 5:** FCSHP and DSI scale scores of patients according to the characteristics of their coexisting diseases

Characteristics	FCSHP Knowledge *Median (Q_1_-Q_3_)*	FCSHP Behaviour *Median (Q_1_-Q_3_)*	FCSHP Attitude *Median (Q_1_-Q_3_)*	FCSHP Total *Median* *(Q1-Q3)*	DSI Total X ± *SS*
**Additional chronic** **disease** Yes No	19.0 (17.0–21.0) 21.0 (19.0–21.0)	21.0 (19.0–23.0) 22.0 (19.0–25.0)	9.0 (8.0–9.0) 9.0 (8.0–10.0)	49.0 (46.0–52.0) 51.0 (47.0–53.0)	66.41 ± 21.00 55.77 ± 24.40

*p*	**<0.001**	0.275	0.275	**<0.001**	**<0.001**

**CRF diagnosis duration** < 3 years 3–8 years 9–14 years ≥ 15 years	19.0 (16.0–21.0)^a^ 20.0 (17.0–21.0)^b^ 19.0 (17.0–21.0)^a,b^ 19.0 (17.0–21.0)^a,b^	21.5 (20.0–25.0) 21.0 (19.0–23.0) 21.0 (19.0–23.0) 22.0 (19.0–23.0)	9.0 (8.0–9.0) 9.0 (8.0–9.0) 9.0 (8.0–9.0) 9.0 (8.0–10.0)	49.0 (45.0–52.0) 49.0 (46.7–52.0) 48.0 (45.0–52.0) 49.0 (45.7–52.0)	61.1 ± 20.84 66.00 ± 21.52 64.79 ± 22.82 67.70 ± 21.41

*p*	**0.029**	0.099	0.871	0.432	0.141

**Hemodialysis duration** < 2 years 2–5 years 6–9 years ≥ 10 years	19.0 (17.0–21.0) 20.0 (18.0–21.0) 19.0 (17.0–21.0) 19.0 (17.0–21.0)	23.0 (20.0–25.0)^a^ 21.0 (19.0–23.0)^b^ 21.0 (19.0–23.0)^b^ 21.0 (18.0–23.0)^b^	9.0 (8.0–9.0) 9.0 (8.0–9.0) 9.0 (8.0–9.0) 9.0 (8.0–10.0)	50.0 (46.5–52.6) 49.0 (46.0–52.0) 49.0 (45.0–52.0) 48.0 (45.0–51.0)	59.81 ± 21.51^a^ 66.70 ± 20.95^b^ 65.90 ± 23.46^b^ 66.69 ± 22.64^b^

*p*	0.370	**<0.001**	0.859	0.086	**0.024**

**Getting information** **about diet and fluid** **restriction** Yes No	21.0 (18.0–21.0) 18.0 (16.0–19.0)	21.0 (19.0–23.0) 22.0 (19.0–23.0)	9.0 (8.0–10.0) 9.0 (7.0–9.0)	49.0 (46.0–52.0) 48.0 (45.0–51.0)	64.31 ± 22.17 66.71 ± 20.70

*p*	**<0.001**	0.118	0.075	**<0.001**	0.208

**Daily fluid intake** < 1000 ml 1000–1500 ml > 1500 ml	19.0 (17.0–21.0)^a^ 19.0 (17.0–21.0)^a,b^ 21.0 (18.0–21.0)^b^	22.0 (19.5–24)^a^ 21.0 (19.0–23.0)^a,b^ 21.0 (19.0–23.0)^b^	9.0 (8.0–9.0) 9.0 (7.0–9.0) 9.0 (9.0–10.0)	49.0 (46.0–52.0) 49.0 (46.0–51.0) 49.0 (45.7–52.0)	65.31 ± 21.28 64.55 ± 21.60 65.28 ± 22.49

*p*	**<0.001**	**0.013**	0.246	0.422	0.928

a,b,c: Co mparisons between measurements in each group

## Discussion

Hemodialysis patients experience many symptoms negatively affecting the quality of life. In this study, the most common symptoms of patients were tiredness or decreased energy, numbness and tingling of feet, difficulty in sleeping and maintainance of sleepiness, respectively. Similar to our study, other studies with HD patients reported that the most common symptoms were fatigue, energy deficiency and sleeping problems^17^,^26^. Fatigue affects the quality of life negatively in patients undergoing hemodialysis treatment^27^. It is thought that most of the patients' fatigue may be due to being elderly, additional chronic disease, and low adherence with fluid restriction. Hemodialysis patients often have difficulties in adherence with treatment. The most difficult and stressful situation is the fluid restriction which makes it difficult for patients to adjust with treatment^9^,^10^. The FCSHP scale was used in this study to evaluate adherence with fluid intake of HD patients. The mean total score of FSCHP scale was 48.68 ± 4.43; mean knowledge, behaviour and attitude subscale scores were 18.85 ± 2.24, 21.28 ± 3.23 and 8.54 ± 1.56, respectively. In a different study, Balım et al. (2013) found that the mean total score of FCSHP scale was 43.88 ± 4.83, and knowledge, behaviour and attitude subscale scores were 8.95 ± 1.81, 22.34 ± 3.64 and 12.57 ± 2.66, respectively^28^. Similarly in Başer and Mollaoğlu's study (2019) found that the mean total score of FCSHP scale was 50.08±5.81, and knowledge, behaviour and attitude subscale scores were 17.74±2.91, 21.08±3.70, 11.16±2.57 respectively^29^.

In the study of Balım (2013)^28^, it was seen that FCSHP total score and knowledge, behaviour and attitude subscale scores were lower than the scores in our study. This may be due to the fact that the educational status of the patients in the study group was higher.

In our study, patients ≤49 years were found to have higher FCSHP total and knowledge subscale scores than others (p<0.001). Likewise, Ahrari et al. found that younger individuals who were treated for HD had higher adherence to treatment^30^. Unlike this study, Efe was found that young people in the 21–35 age group undergoing HD treatment had lower adherence with fluid restriction than other age groups^9^. The reason for having higher FCSHP scale scores of young people may be higher education levels than other age groups, so they may have information easier and have better adherence with recommended treatment.

Supporting the spouses of individuals undergoing HD treatment relieves the patient psychologically and helps them cope with healyh problems more easily^9^. In this study, married patients were found to have higher FCSHP knowledge subscale and total scores (p<0.05). Similar to our results, Günalay et al. stated that married patients had higher adherence with treatment^31^. Being married is thought to facilitate adherence with fluid control^9^. This may be due to the fact that married HD patients share the basic needs with their family, families support them and so they can manage the symptoms more easily and effectively.

Factors such as the presence of a chronic disease, dependence on a machine and labour loss make it difficult for patients to adapt to treatment and cause intense stress^32^. In the study, 87.4% of HD patients were found to have another chronic disease in addition to renal failure. Patients without additional chronic disease were found to have higher FCSHP knowledge subscale and total scores (p<0.001) According to these results, it can be said that the patients with no additional chronic diseases are better than the others in adherence with fluid control. DSI scores of patients with additional chronic disease were found to be higher in our study (p<0.001). Additional chronic diseases affect many different systems, leading to more symptoms.

Being educated about the disease provides prevention of the possible health problems, improvement of the quality of life and facilitation of the self-care^33^. Patients who were informed about diet and fluid restriction were found to have higher FCSHP knowledge subscale and total scores (p<0.05).

Being informed about the disease and fluid control shortens the time to adapt to fluid control. Baraz et al. have shown that education of HD patients about their disease and management increases the adherence with treatment^34^. Regular education of patients about treatment process, medication, diet, fluid intake and disease management affects the adherence with treatment positively^35^.

Interdialytic weight gain is caused by daily water and salt intake between two dialysis sessions. Daily fluid intake determines the interdialytic weight of patients and various problems occur in patients with interdialytic weight over 2–3 kg. One of the most important problems that can be seen in patients with high interdialytic weight is hypertension (HT)^34^. In this study, patients with daily fluid intake less than 1000 ml were found to have higher FCSHP knowledge and behaviour subscale scores (p<0.05). The fact that patients are more knowledgeable about fluid control provides a reduction in daily fluid intake. Similarly, Kurt et al. were found a negatively significant relationship between daily fluid intake and fluid control. It has been emphasized that as the patients' fluid intake decreases, their adherence with fluid control increases^36^.

The condition causing the most common problems in HD patients and complicates treatment adherence is fluid restriction. The inability of HD patients to provide fluid control can lead to some complications, a decrease in quality of life and a threat to patients' safety^9^,^10^. In this study, there was a negative correlation between FCSHP subscale, total and DSI scores of HD patients (p<0.001). Patients' problems with fluid restriction can lead to HT, pulmonary edema, left ventricular failure and prematüre mortality^16^,^17^. As these problems increase, many symptoms such as fatigue, muscle cramps and sexual dysfunction occur^16^.

## Limitation of study

Since the study was conducted in one geographical region the results may not represent hemodialysis patients from other regions. Due to this limitation, the results of the study cannot be generalized.

## Conclusion

According to the data obtained from the study, the most common symptoms experienced by patients are feeling tired or decreased energy, numbness-tingling in the feet, difficulty in falling asleep and difficulty in maintaining sleep. It was determined that the patients experienced less symptoms as their compliance with fluid control increased.

According to these results, regular evaluation of adherence with fluid control and the symptoms of patients undergoing HD treatment, planning nursing attempts about factors that cause incompatibilty, training and counselling of the risk groups having difficulties about adherence with fluid control, and conducting more comprehensive experimental studies for common symptoms (fatigue, insomnia) of patients with HD treatment can be suggested.
